# Long-term outcomes after allogeneic hematopoietic stem cell transplantation for metachromatic leukodystrophy: the largest single-institution cohort report

**DOI:** 10.1186/s13023-015-0313-y

**Published:** 2015-08-07

**Authors:** Alexander A. Boucher, Weston Miller, Ryan Shanley, Richard Ziegler, Troy Lund, Gerald Raymond, Paul J. Orchard

**Affiliations:** Department of Internal Medicine and Pediatrics, University of Minnesota, Minneapolis, MN 55455 USA; Division of Pediatric Blood and Marrow Transplantation, 420 Delaware Street SE, MMC 484, Minneapolis, MN 55455 USA; Biostatistics Core, Masonic Cancer Center, University of Minnesota, Minneapolis, MN 55455 USA; Division of Pediatric Clinical Neurosciences, University of Minnesota, Minneapolis, MN 55455 USA

**Keywords:** Metachromatic leukodystrophy, Hematopoietic stem cell transplantation, Umbilical cord blood transplantation, Bone marrow transplantation

## Abstract

**Background:**

Metachromatic Leukodystrophy (MLD) is a rare, fatal demyelinating disorder with limited treatment options. Published outcomes after hematopoietic stem cell transplantation (HSCT) are scant and mixed. We report survival and function following HSCT for a large, single-center MLD cohort.

**Methods:**

Transplant-related data, survival and serial measures (brain MRI, nerve conduction velocity (NCV), neurologic and neuropsychology evaluations) were reviewed. When possible, parental interviews informed current neurologic status, quality-of-life, and adaptive functioning. Gross motor and expressive functions for late-infantile (LI-MLD) and juvenile (J-MLD) patients were described using previously reported, MLD-specific scales.

**Results:**

Forty patients with confirmed MLD have undergone HSCT at our center. Twenty-one (53 %) survive at a median 12 years post-HSCT. Most deaths (n = 17) were treatment-related; two died from disease progression. Survival did not depend upon MLD subtype or symptom status at transplant. LI-MLD patients survive beyond reported life expectancy in untreated disease.

Abnormal brain MRI and peripheral nerve conduction velocities (NCV) were common before HSCT. Following transplant, fewer patients experienced MRI progression compared to NCV deterioration.

Sixteen LI-MLD and J-MLD survivors were evaluable for long-term gross motor and/or expressive language functioning using existing MLD clinical scoring systems. While most J-MLD patients regressed, the aggregate cohort demonstrated superior retention of function compared to published natural history.

Seventeen LI-MLD, J-MLD and adult subtype (A-MLD) survivors were evaluable for long-term adaptive functioning, activities of daily living, and/or cognition. Relative cognitive sparing was observed despite overall global decline.

Five sibling pairs (one LI-MLD and four J-MLD), in which at least one underwent transplant in our cohort, were evaluable. Within each familial dyad, survival or function was superior for the treated sibling, or if both siblings were transplanted, for the pre-symptomatic sibling.

**Conclusions:**

HSCT is a viable treatment option for MLD, but has significant limitations. Later-onset phenotypes may benefit most from early, pre-symptomatic transplant. Until superior, novel treatment strategies are demonstrated, MLD patients should be carefully considered for HSCT.

## Background

Metachromatic leukodystrophy (MLD) is a rare, fatal, demyelinating lysosomal storage disease usually caused by deficient arylsulfatase A (ARSA) enzyme activity. An autosomal recessive disorder, MLD results from pathologic excess of cerebroside sulfatase (sulfatide), a major lipid component of myelin [1]. Sulfatide accumulation eventually leads to central and peripheral myelin destruction, producing the clinical and radiologic signs characteristic of MLD [2]. If MLD is suspected, the diagnosis is typically made upon finding a combination of low tissue ARSA activity and excessive urinary sulfatides. Confirmation is often achieved by detecting causative *ARSA* gene mutations.

Three distinct clinical subtypes of MLD are recognized based on the age of disease manifestation: late-infantile (LI-MLD, around 2 years of age), juvenile (J-MLD, ages 3-16 years), and adult (A-MLD, ages >16 years) [3] While MRI findings are generally consistent across phenotypes, the neurobehavioral changes vary by subtype [4]. LI-MLD and occasionally J-MLD can present with rapid global neurologic regression over months and result in early death without treatment. Neurologic findings in these earlier-onset phenotypes are primarily motor related, including weakness, gait abnormalities, quadriparesis, dysarthria, hearing difficulties, vision impairment, and incontinence [5]. In contrast, patients with later onset of disease may present with a neuropsychiatric or cognitive prodrome, including frontal lobe dysregulation, which can be followed by gradual but frank neurologic decline [6].

Treatment options for MLD remain limited. Hematopoietic stem cell transplantation (HSCT) has been used for decades on the basis of providing metabolic cross-correction, in which functional ARSA from donor-derived cells promotes sulfatide degradation. However, reports of transplant outcomes in the medical literature are relatively few and conclusions are mixed [7–17].

Therefore, we performed a single-center, retrospective review of long-term outcomes following HSCT for patients diagnosed with MLD. We aimed to describe survival, transplant-related, neuroradiographic, neurophysiologic, gross motor, expressive language, neurocognitive and quality-of-life outcomes. This report, believed to be the largest known of its kind, aims to more clearly illuminate the longitudinal functional and clinical outcomes after HSCT.

## Methods

### Cohort identification, patient-related data and transplant-related measures

All patients undergoing HSCT for the diagnosis of MLD were identified from the prospectively maintained University of Minnesota Blood and Marrow Transplant Database (BMT Database). Those whose MLD diagnosis could be retrospectively confirmed were included for this analysis. MLD diagnosis was considered confirmed if (1) the patient had low ARSA with elevated urine sulfatides (n = 39); or (2) the patient had low ARSA, radiographic and clinical evidence of leukodystrophy and an affected sibling with low ARSA and elevated urine sulfatides (n = 1). For each patient, data were collected from the BMT Database, medical record review, and/or parental telephone surveys (approved by the Institutional Review Board [IRB] and following the provision of informed consent). All efforts were made to extract the following information per patient: age at clinical MLD onset, reason for diagnosis, family history of MLD, age at HSCT, transplant conditioning intensity, allograft source, donor-recipient human leukocyte antigen (HLA) compatibility, occurrence and time to neutrophil and platelet recovery, occurrence and severity of acute and chronic graft-versus-host disease (aGvHD, cGvHD) [18, 19], most recent post-transplant donor hematopoietic engraftment, most recent leukocyte ARSA activity, survival status, cause of death (where applicable), and time to most recent follow-up or death.

Though MLD diagnosis was assigned to each patient prior to transplant by the evaluating neurologist and BMT physician, available records were also reviewed for confirmation of diagnosis. Baseline (pre-transplant) leukocyte ARSA activity and urine sulfatide excretion were noted to be primary source (laboratory report available for review) or secondary source (laboratory result referenced in the medical record). Medical histories and examinations were reviewed for dermatologic or musculoskeletal findings consistent with multiple sulfatase deficiency. MLD subtype assignment was retrospectively assigned by age when disease became clinically evident in a patient or sibling proband(s) according to the medical record. Subtype assignment followed criteria used in the largest published natural history study to date: LI-MLD disease, < 30 months; J-MLD disease, 30 months to 15 years; A-MLD disease, ≥ 16 years [20]. Patients were defined as symptomatic if they demonstrated any clinical evidence of MLD at the time of transplant.

All patients were treated in accordance with the Declaration of Helsinki on protocols approved by the University of Minnesota IRB. Patients or guardians provided informed consent for treatment and publication of outcome data. Conditioning regimens and hematopoietic stem cell allografts were selected according to institutional algorithms at the time of transplantation. HLA typing of donors and recipients was by allele- or antigen-based methods, depending upon institutional guidelines at the time. All sibling donors were screened for leukocyte ARSA activity and determined for carrier status by the treating BMT physician. Neutrophil recovery was defined as the first of 3 consecutive days with a peripheral blood neutrophil count ≥ 500/μL. Platelet recovery was defined as the first of 7 consecutive days with a transfusion-independent peripheral platelet count ≥ 20 x 10^3^/μL. Most recent donor hematopoietic engraftment was determined according to existing institutional guidelines on either marrow-aspirated nucleated cells, unfractionated peripheral blood leukocytes, or myeloid-enriched peripheral blood leukocytes. Most recent leukocyte ARSA activity was determined by clinical laboratory measurement. Each patient underwent GvHD prophylaxis according to existing protocol. Infectious disease prophylaxis, growth factor administration, and blood product support were per University of Minnesota BMT Program standard of care guidelines.

### Gross motor and expressive language function evaluation

**Fig. 1:**
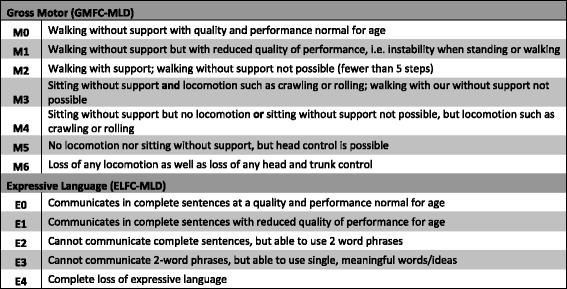
Gross Motor Function Classification (GMFC-MLD) and Expressive Language Function Classification (ELFC-MLD) scales in metachromatic leukodystrophy. The previously reported scales describe both gross motor and expressive language deterioration over time for late-infantile and juvenile subtypes of MLD. Long-term survivors in the HSCT cohort were evaluated for scores at most recent follow-up and age-to-entry into various levels over time

Longitudinal gross motor and expressive language functioning over time for LI-MLD and J-MLD long-term survivors were assessed with the Gross Motor Function Classification for MLD (GMFC-MLD) and Expressive Language Function Classification for MLD (ELFC-MLD) scales, as previously reported (Fig. 1) [20–22]. The age at entry into a given level on both scales was tracked for each patient. When possible, current and retrospective GMFC-MLD and ELFC-MLD data were obtained via parental telephone survey using the methodology described by Kehrer [20]; otherwise, most recent scores and ages at entry into levels were constructed through review of detailed clinical neurology assessments in the medical record.

### Brain magnetic resonance imaging and peripheral nerve conduction velocity studies

Clinical brain MRI reports at pre-HSCT and post-HSCT time points were reviewed as available for all MLD subtypes. Each report was characterized according to the presence or absence of pathologic white matter findings and whether changes relative to the previous scan (worse, stable, or better) were noted by the interpreting neuroradiologist. The presence or absence of atrophy was not considered when assessing serial MRI reports, as inconsistencies in reporting volume loss were noted upon review of records. Similarly, clinical nerve conduction velocity (NCV) reports from pre-HSCT and post-HSCT time points were reviewed as available for all cohort members. Each report was assessed for the presence or absence of abnormalities and whether general changes relative to the previous study (worse, stable, or better) were noted by the interpreting neurologist.

### Neuropsychologic, adaptive behavior functioning and quality-of-life assessments

Neuropsychology records of all long-term cohort survivors were reviewed for assessment of cognition and adaptive behavior functioning at all available pre-HSCT and post-HSCT time points. Cognition was trended longitudinally with standardized Verbal Intelligence Quotient (VIQ) scores by Wechsler-series assessments [23]. In rare instances when VIQ data was not available at a time point, Wechsler Full-Scale scores (FSIQ, n = 1) or equivalent domain scores on the Stanford-Binet (n = 1) or Bayley Scales of Infant Development (n = 2) tools were used [24, 25]. Similarly, age equivalent composite adaptive behavior function as assessed by the Vineland Adaptive Behavior Scales (VABS) was trended over time for all long-term survivors [26]. When possible, current VABS scores were generated for this analysis via parental telephone survey (n = 14).

We further used recent parental surveys to assess several other outcomes and attitudes following HSCT for all subtypes of MLD. First, long-term survivor quality-of-life was measured by the Cornell-Brown Scale (CBS) [27]. Next, the age at loss of independent performance of common activities of daily living (ADLs) was determined. Finally, parental attitude regarding satisfaction with having chosen HSCT for their child with MLD was assessed.

### Comparison of transplant outcomes and natural history for MLD sibling pairs

When available, long-term survival and functional outcome data (GMFC-MLD, ELFC-MLD, VABS and ADL) between sibling pairs with MLD were evaluated for descriptive purposes. For some pairs, only one sibling underwent HSCT at our center. Data for non-transplanted siblings (n = 3), or siblings transplanted at another center (n = 1) were obtained by parental telephone survey.

### Statistical analyses

Statistical analyses were descriptive and used standard summary measures such as frequencies, medians, and interquartile ranges. Overall survival from time of HSCT was calculated using the Kaplan-Meier method [28]. The cumulative incidence function with competing risks was used to estimate transplant-related mortality (TRM; competing risk = death from MLD progression), along with neutrophil recovery, platelet recovery, and GvHD (competing risk = death) [29].

## Results

### Cohort characteristics, survival and transplant-related outcomes

Forty-three patients underwent HSCT for a reported diagnosis of MLD at the University of Minnesota with transplant dates spanning June 1984 to April 2013 (Table 1). Forty patients (93 %) in whom diagnosis could be confirmed were ultimately considered for analysis. Twelve patients (30 %) were male. Four patients (10 %) had LI-MLD, 27 (67 %) had J-MLD, and 9 (23 %) had A-MLD. Five patients (12 %) underwent reduced-intensity conditioning (RIC) using melphalan, clofarabine, low-dose total-body irradiation (TBI) and alemtuzumab. The remaining 35 (88 %) received myeloablative conditioning that was either busulfan/cyclophosphamide (Bu/Cy) or cyclophosphamide/TBI (Cy/TBI)-based. Eleven patients (27 %) received related-donor marrow, 14 (35 %) received unrelated-donor marrow, and 15 (38 %) received umbilical cord blood (UCB) transplants. In four patients (10 %), the sibling donor was considered a carrier due to low leukocyte ARSA activity, though full cataloging of parental and sibling ARSA activities and genotyping (for the exclusion of possible pseudodeficiency allele carriage to explain ARSA hypo-activity) was not performed [30]. Three patients underwent a second transplantation due to autologous hematopoietic recovery: Patient ID9 at 4 months after first HSCT, ID5 at 18 months, and ID11 at 6 months.

**Table 1:** Patient demographics, transplant-related and survival characteristics

Cohort ID	Gender	Time from symptom onset to HSCT (y,m)	Age at HSCT (y,m)	Transplant Year	Conditioning regimen	Donor type	HLA matching	% Donor engraftment (y,m)^c^	% ARSA Activity (y,m)^c^	Time to follow-up (y,m)^c^	Cause of death
Late infantile											
1	M	X	0,4	1996	MA (Bu/Cy)	cRD	6/6	100 (0,4)	—	0,3^a^	VOD
2	M	X	0,5	2004	MA (Bu/Cy)	UCB	5/6	100 (2,11)	100 (2,10)	4,10	
3	F	X	0,8	1995	MA (Bu/Cy)	cRD	6/6	100 (8,4)	60 (6,10)	19,6	
6	F	0,6	2,10	1995	MA (Bu/Cy)	URD	5/6	100 (1,6)	100 (1,0)	1,6^a^	unknown
Juvenile											
4	F	1,10	4,10	1984	MA (Bu/Cy)	RD	6/6	100 (15,1)	100 (25,2)	30,6	
9	F	1,2	4,2^b^	2001	MA (Cy/TBI)	URD	6/6	100 (4,9)	100 (3,5)	13,3	
5	F	X	1,3^b^	1994	MA (Bu/Cy)	URD	6/6	100 (0,3)	—	2,2^a^	cGvHD
7	F	X	2,8	1994	MA (Bu/Cy)	URD	6/6	100 (0,2)	—	0,3^a^	VOD
8	F	X	3,5	1994	MA (Bu/Cy)	URD	6/6	97 (6.5)	100 (6,5)	8,2	
22	F	X	11,1	2004	MA (Bu/Cy)	dUCB	4/6 + 4/6	100 (3,2)	100 (0,6)	10,7	
10	F	0,5	5,8	1996	MA (Cy/TBI)	URD	6/6	60 (11,3)	38 (11,3)	18,7	
11	F	0,10	5,10^b^	1998	MA (Cy/TBI)	cRD	5/6	100 (2,6)	25 (2,6)	7,2^a^	MLD
12	F	1,1	5,11	2008	RIC	UCB	6/6	2 (0,1)	8 (0,1)	3,1^a^	MLD
13	F	0,11	6,4	1989	MA (Bu/Cy)	RD	6/6	—	73 (0,1)	0,2^a^	MSOF
14	F	2,3	6,3	2002	MA (Bu/Cy)	UCB	4/6	100 (2,0)	100 (2,0)	2,0	
15	F	0,0	6,7	1997	MA (Bu/Cy)	URD	6/6	100 (1,7)	100 (1,7)	7,3	
16	F	3,6	7,6	1999	MA (Cy/TBI)	URD	5/6	67 (3,5)	59 (1,9)	15,7	
17	M	2,8	7,8	2003	MA (Bu/Cy)	UCB	5/6	100 (0,6)	100 (0,6)	11,9	
18	M	0,10	8,4	1997	MA (Bu/Cy)	URD	6/6	100 (0,7)	100 (0,6)	0,7^a^	Sepsis
19	M	0,9	8,7	2002	MA (Bu/Cy)	UCB	5/6	100 (0,5)	100 (0,5)	10,6^a^	cGvHD
20	F	2,4	11,1	2005	MA (Bu/Cy)	dUCB	4/6 + 4/6	100 (0,3)	100 (0,3)	0,3^a^	Sepsis
21	F	2,0	10,4	1995	MA (Bu/Cy)	RD	6/6	100 (0,1)	—	0,2^a^	aGvHD
23	M	2,7	11,6	1991	MA (Bu/Cy)	URD	6/6	—	50 (0,7)	0,7^a^	Sepsis
24	F	1,5	14,8	1989	MA (Bu/Cy)	RD	6/6	100 (16,4)	100 (16,4)	16,4	
25	F	1,7	16,4	2004	MA (Bu/Cy)	dUCB	6/6 + 6/6	100 (0,4)	100 (0,2)	0,5^a^	TTP
26	F	1,10	16,2	1994	MA (Bu/Cy)	cRD	6/6	100 (9,5)	100 (11,0)	12,6	
27	F	5,1	16,1	2004	MA (Bu/Cy)	UCB	4/6	100 (3,2)	100 (3,2)	10,7	
28	M	3,11	16,5	2002	MA (Bu/Cy)	UCB	4/6	100 (0,4)	100 (0,4)	0,8^a^	Sepsis
30	M	10,5	20,5	2000	MA (Cy/TBI)	URD	5/6	100 (0,2)	100 (0,2)	0,6^a^	Sepsis
32	F	16,3	27,7	1996	MA (Cy/TBI)	RD	6/6	44 (2,0)	32 (2,0)	18,2	
43	M	5,8	19,8	2013	RIC	RD	6/6	100 (1,1)	100 (1,1)	1,6	
Adult											
42	F	X	20,1	1995	MA (Bu/Cy)	URD	5/6	100 (0,1)	—	0,1^a^	VOD
31	M	9,1	27,1	2003	MA (Bu/Cy)	UCB	4/6	100 (0,2)	—	0,3^a^	Graft Failure
33	F	10,10	29,10	1999	MA (Cy/TBI)	URD	6/6	100 (3,0)	100 (3,0)	10,0	
34	M	3,7	33,7	2000	MA (Cy/TBI)	URD	5/6	100 (1,5)	64 (0,1)	10,0	
36	F	5,4	38,1	1999	MA (Cy/TBI)	RD	6/6	35 (2,3)	76 (1,0)	1,1	
38	F	0,11	40,7	2001	MA (Cy/TBI)	UCB	5/6	100 (0,3)	100 (0,3)	0,4^a^	Sepsis
39	M	5,5	42,5	2007	RIC	dUCB	5/6 + 5/6	100 (0,6)	100 (1,3)	7,6	
40	F	3,5	42,6	2007	RIC	dUCB	5/6 + 6/6	80 (2,9)	100 (1,2)	6,5^a^	unknown
41	F	3,2	44,2	2007	RIC	dUCB	4/6 + 5/6	0 (1,1)	50 (0,2)	1,1	

*y* Year, *m* Month, *X* Asymptomatic, *MA* Myeloablative, *Bu* Busulfan, *Cy* Cyclophosphamide, *TBI* Total-body irradiation, *RIC* Reduced-intensity conditioning, *RD* Related marrow donor (sibling), *URD* Unrelated marrow donor, *UCB* Umbilical cord blood, *d* Double cord, *c* MLD-carrier marrow donor, ^a^ Deceased, *VOD* Hepatic veno-occlusive disease, *MSOF* Multi-system organ failure, *aGVHD* Acute graft-vs-host disease, *cGvHD* Chronic graft-vs-host disease, ^b^ Age denoted is time of first transplant, ^c^ Most recent time point available, expressed relative to date of HSCT. Patients 29, 35 and 37 were removed due to lack of confirmatory MLD diagnostic data on retrospective review

Thirty-nine patients (98 %) had primary and/or secondary source data documenting baseline low leukocyte ARSA activity and elevated urine sulfatide excretion for confirmation of MLD diagnosis (Table 2). No patient had records of exam findings consistent with multiple sulfatase deficiency. In one patient with low baseline ARSA activity and white matter abnormalities evident on brain MRI, low ARSA and elevated urine sulfatide were documented in an affected sibling. In three separate patients who underwent HSCT for MLD, all with abnormal NCV studies and white matter disease on brain MRI, no urine sulfatide data was found. These patients were excluded from analysis due to the absence of confirmatory testing for MLD. For these 3 patients, neither *ARSA* molecular data nor nervous tissue biopsy data was available for genotypic or histopathologic confirmation of diagnosis.

**Table 2:** MLD-related characteristics

Cohort ID	Age at diagnosis (y,m)	Reason for diagnosis	ARSA activity at HSCT (nmol/hr/mg)	Urine sulfatides Elevated? (μg/mg creatinine)	White matter abnormality on brain MRI at HSCT?	NCV Abnormality at HSCT?	VABS Reported?^!^	VIQ Reported?^!^	Notes
Late infantile									
1	0,1	Fam	Low^a^	Yes (3.4)	No Data	Yes			Sibling proband symptomatic before 2 years old
2	0,1	Fam	3.3	Yes^a^	No	Yes	Yes		Sibling proband symptomatic before 2 years old
3	0,4	Fam	3.0	Yes (4.9)	Yes	Yes	Yes		Sibling proband symptomatic before 2 years old
6	1,3	Fam/SS	Low^a^	Yes (4.8)	Yes	Yes	Yes	Yes	Two sibling probands symptomatic at 2 years old
Juvenile									
4	0,5	Fam	9.2	Yes (2.5)	No Data	Yes	Yes	Yes	
9	3,11	SS	3.9	Yes (Excessive^b^)	Yes	Yes	Yes	Yes	
5	0,10	Fam	Low^a,c^	Yes (5.2)	No Data	Yes	Yes		Sibling proband symptomatic at 3 years old
7	2,2	Fam	Low^a^	Yes (10.3)	Yes	Yes			Sibling proband symptomatic before 8 years old
8	3,1	Fam	2.0	Yes (10.9)	Yes	Yes		Yes	Sibling proband symptomatic before 5 years old
22	10,10	Fam	8.6	Yes (Excessive^b^)	Yes	Yes	Yes	Yes	Sibling proband symptomatic at 11 years old
10	5,2	Fam/SS	5.5	Yes (6.7)	Yes	Yes	Yes	Yes	
11	5,7	SS	3.4	Yes (6.4)	Yes	Yes			
12	5,8	SS	8.8	Yes (5.9)	Yes	No Data			
13	6,0	SS	9.3	Yes (3.6)	Yes	Yes			
14	6,0	SS	8.9	Yes (17.9)	Yes	Yes			
15	6,4	Fam/SS	8.3	Yes (Excessive^b^)	No Data	Yes			
16	7,2	SS	2.7	Yes (7.9)	Yes	Yes	Yes	Yes	
17	7,6	SS	6.7	Yes (9.3)	Yes	Yes	Yes		
18	8,3	Fam/SS	11.2	Yes (Excessive^b^)	No Data	Yes			
19	8,5	SS	15.5	Yes (5.4)	Yes	Yes	Yes		
20	9,9	Fam/SS	1.9	Yes (3.0)	Yes	Yes			
21	10,3	Fam/SS	43.2	Yes (1.1)	Yes	Yes			
23	10,11	SS	10.1	Yes (1.2)	Yes	Yes			
24	14,2	Fam/SS	5.2	Yes (3.1)	Yes	Yes	Yes		
25	15,6	SS	3.6	Yes (0.7)	Yes	Yes			
26	15,9	SS	4 % normal^d^	Yes (2.4)	Yes	Yes	Yes	Yes	
27	15,10	SS	6.8	Yes (Excessive^b^)	Yes	Yes	Yes	Yes	
28	15,11	SS	7.7	Yes (3.2)	Yes	Yes			
30	19,8	SS	6.0	Yes (Excessive^b^)	Yes	Yes			
32	26,4	SS	35.1	Yes (Excessive^b^)	Yes	Yes	Yes		
43	19,2	SS	4.1	Yes (Excessive^b^)	Yes	Yes			
Adult									
42	20,1	Fam	7.9	Yes (1.3)	Yes	Yes			Two older siblings reportedly with MLD
31	26,0	SS	3.7	Yes (3.8)	Yes	No			
33	28,10	SS	8.7	Yes (3.5)	Yes	Yes		Yes	
34	32,0	Fam/SS	10.3^a^	No Data	Yes	No			Sibling of Patient ID 33
36	35,9	SS	3.5^a^	Yes^a^	Yes	No	Yes	Yes	
38	40,0	Fam/SS	5.9	Yes (8.2)	Yes	Yes			
39	42,0	SS	10 % normal^d^	Yes^a^	Yes	Yes	Yes	Yes	
40	42,1	Fam/SS	Low^a^	Yes (6.6)	Yes	Yes			
41	43,10	SS	21.8	Yes (6.6)	Yes	Yes	Yes	Yes	

*y* Year, *m* Month, *!* Denotes if patient included for long-term adaptive behavior functioning or cognition in this analysis, *Fam* Family history, *SS* Signs or symptoms of MLD, ^a^ Primary laboratory result not found; results as stated in medical record, ^b^ Primary laboratory result reported qualitatively only, ^c^ Additional primary result of 16.0 nmol/hr/mg following HSCT with donor hematopoietic engraftment of 25 %, ^d^ Primary laboratory result reported as percentage of normal or converted to such. Patients 29, 35 and 37 were removed due to lack of confirmatory MLD diagnostic data on retrospective review

**Fig. 2:**
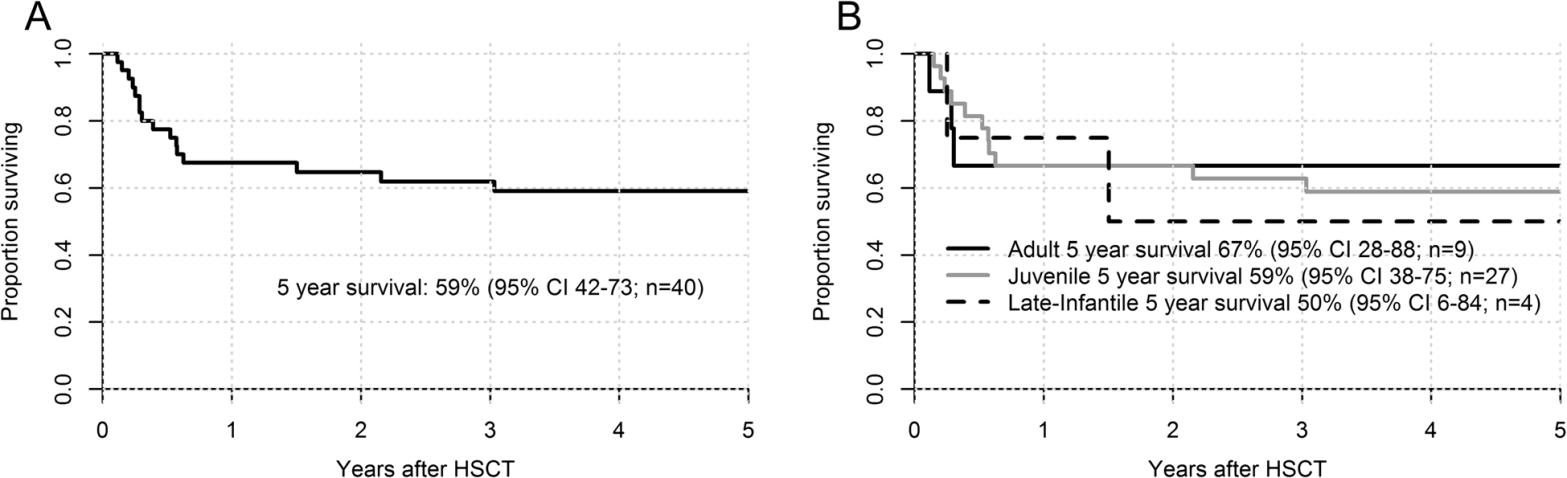
Kaplan-Meier survival estimates following HSCT for MLD. **a** Probability of survival at 5 years following HSCT for the entire cohort (n = 40). **b** Probability of survival at 5 years following HSCT for individual MLD subtypes: LI-MLD (n = 4); J-MLD (n = 27), and A-MLD (n = 9)

Twenty-one patients (53 %) are alive at a median post-transplant follow-up of 10 years. For the entire cohort, the Kaplan-Meier (KM) estimate of survival at 5 years is 59 % (95 % confidence interval [CI95], 42 % - 73 %; Fig. 2). For each MLD subtype, the KM estimate of survival at 5 years was as follows: LI-MLD, 50 % (CI95, 6 % - 84 %); J-MLD, 59 % (CI95, 38 % - 75 %); A-MLD, 67 % (CI95, 28 % - 88 %; Fig. 2). For the entire cohort, survival was independent of the conditioning regimen, MLD subtype, and the presence of symptoms at the time of transplantation. A trend toward inferior survival was noted for recipients of unrelated marrow allografts, as compared to those who underwent related-donor or UCB transplantation.

The cumulative incidence of neutrophil recovery for the entire cohort was 95 % (CI95, 84 % - 99 %) at a median day +16 (CI95, days 12 - 17). The cumulative incidence of platelet recovery was 80 % (CI95, 58 % - 96 %) at a median day +42 (CI95, days 33 - 53). The cumulative incidence of Grade II-IV aGvHD was 44 % (CI95, 26 % - 62 %) and Grade III-IV aGvHD was 16 % (CI95, 4 % - 29 %). The cumulative incidence of any cGvHD was 32 % (CI95, 14 % - 50 %). The cumulative incidence of TRM by day +180 was 23 % (CI95, 12 % - 36 %). Five patients experienced primary autologous hematopoietic recovery, 2 of these (40 %) following RIC.

### Gross motor and expressive language function outcomes

**Fig. 3:**
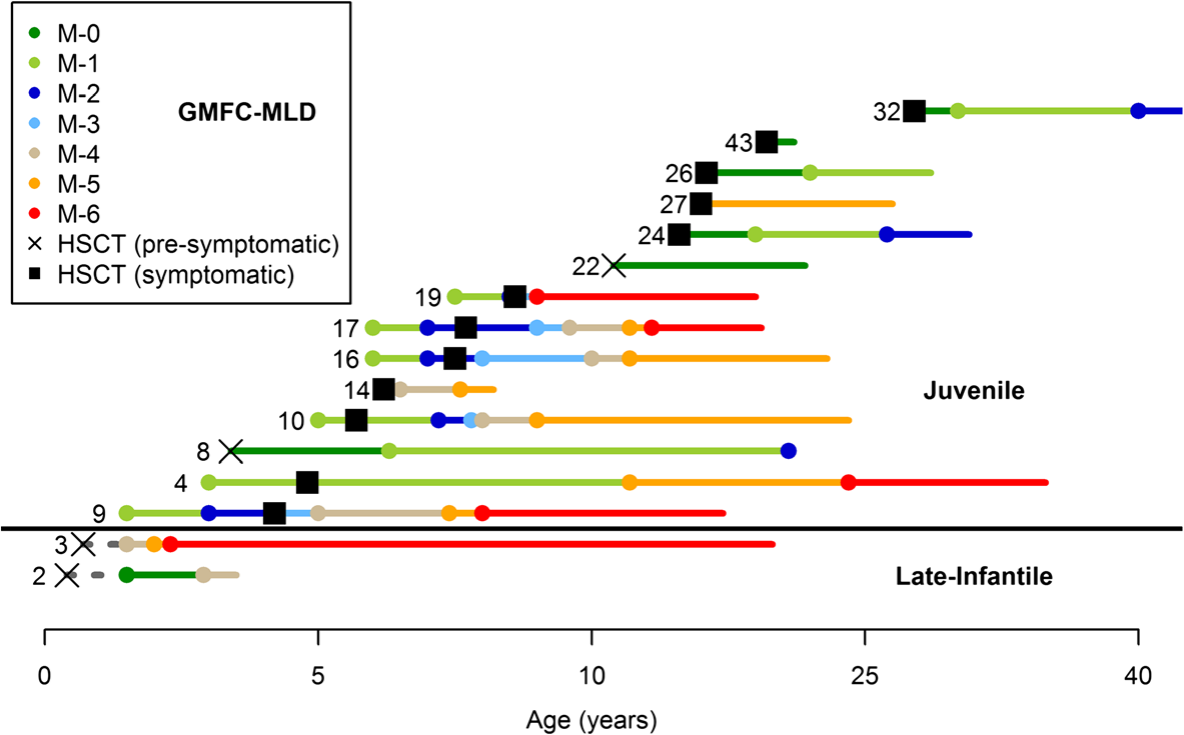
Age to entry into GMFC-MLD levels for LI-MLD and J-MLD patients in the HSCT cohort. Numbers preceding each line refer to the Patient ID (see Tables 1 and 2). Circles represent time at entry into a respective level. Each line ends at most recent follow-up. Some patients had evaluable gross motor function data prior to HSCT. See Fig. 1 for GMFC-MLD level definitions. Patients were labeled symptomatic at the time of HSCT if they exhibited any clinical manifestation of MLD

**Fig. 4:**
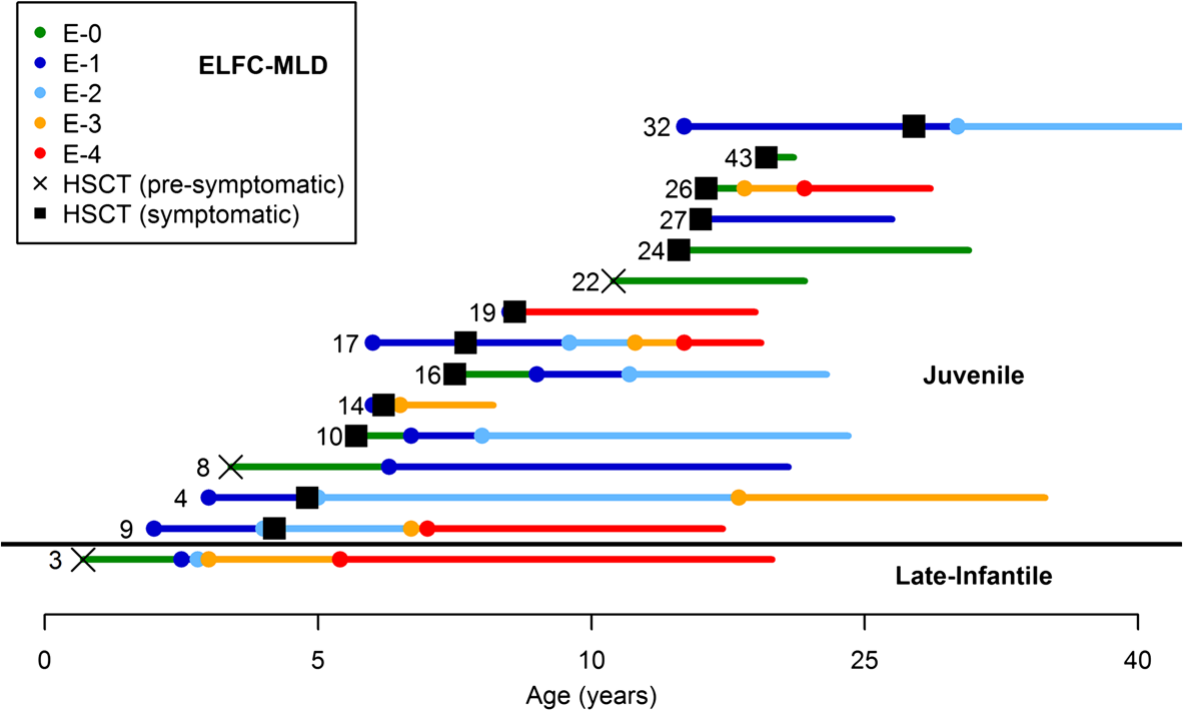
Age to entry into ELFC-MLD levels for LI-MLD and J-MLD patients in the HSCT cohort. Numbers preceding each line refer to the Patient ID (see Tables 1 and 2). Circles represent time at entry into a respective level. Each line ends at most recent follow-up. Some patients had evaluable expressive language function data prior to HSCT. See Fig. 1 for ELFC-MLD level definitions. Patients were labeled symptomatic at the time of HSCT if they exhibited any clinical manifestation of MLD

Sixteen long-term survivors with either LI-MLD (n = 2) or J-MLD (n = 14) were evaluable for longitudinal gross motor and/or expressive language functioning by the GMFC-MLD and ELFC-MLD scales, respectively (Figs. 3 and 4). The median post-HSCT time to most recent evaluation was 12 years, 5 months, while the median age to most recent evaluation was 21 years, 6 months. For 11 of these patients, performance on the scales was determined by recent telephone interview. For the remaining 5 patients, scores were constructed using clinical neurology assessments in the medical record.

### Brain MRI and peripheral NCV outcomes

**Fig. 5:**
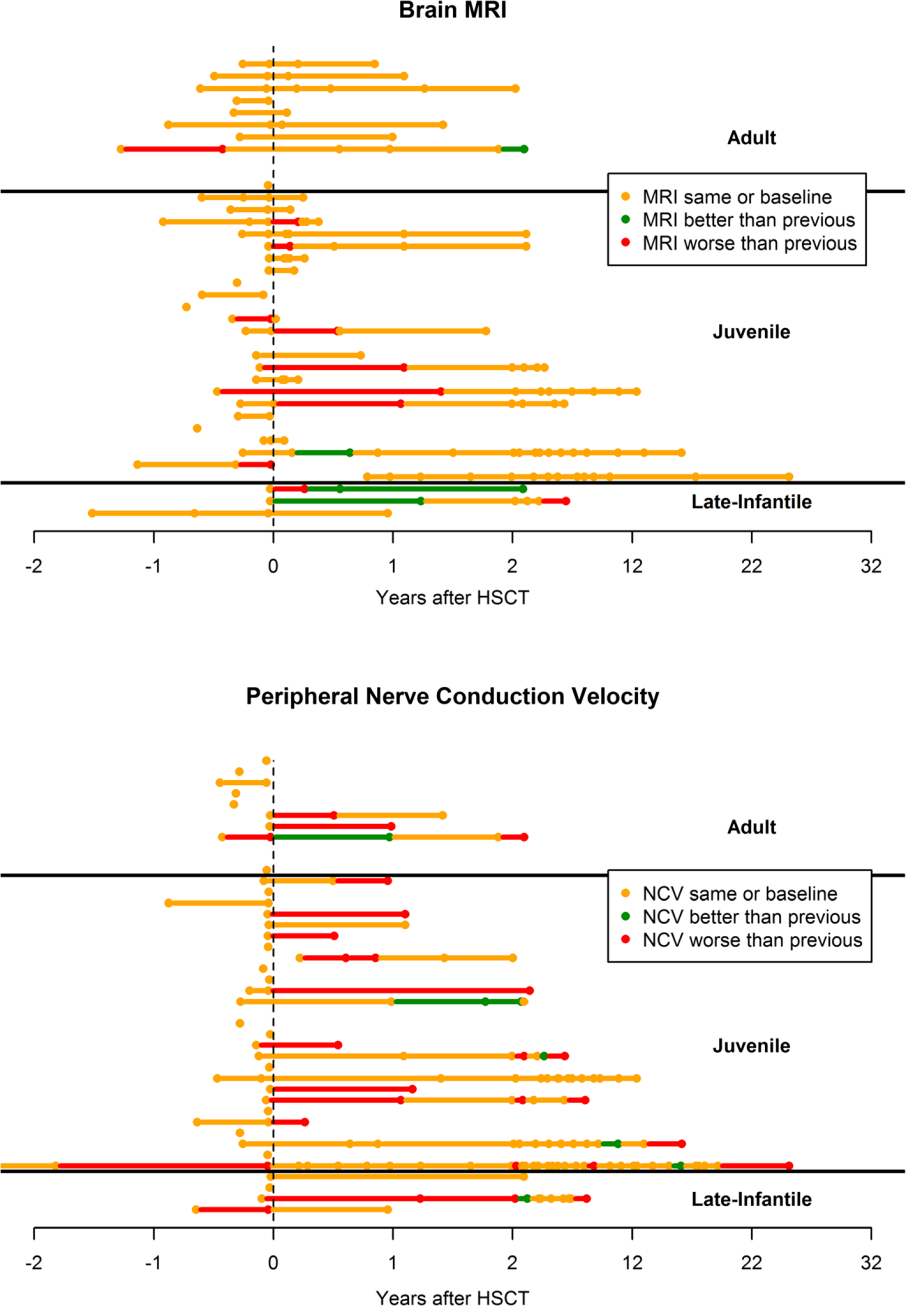
Brain MRI and NCV trends over time. All available clinical reports of brain MRI and peripheral nerve conduction velocity studies for the cohort were reviewed. Each MRI time-point was evaluated for change in white matter disease from the previous study as classified by the interpreting neuroradiologist. Each NCV time-point was evaluated for change in function from the previous study as classified by the interpreting neurologist. Each line represents a unique patient, while each circle represents an assessment. The dashed vertical line at time 0 reflects the time of transplant. Negative time points denote pre-HSCT studies. Red, yellow and green circles and preceding line segments indicate MRI or NCV studies that were worse than, the same as, or better than the previous assessment, respectively. Of evaluable patients at pre-transplant baseline, 97 % had an abnormal MRI while 92 % demonstrated peripheral neuropathy

Thirty-five patients (88 %) had at least one pre-HSCT brain MRI report available for review (Fig. 5). For 34 of these patients (97 %), baseline abnormal white matter was observed. Twenty-three patients (66 %) had at least one post-HSCT study beyond day +60 with a median time to last follow up of 23 months (Interquartile Range (IQR), 9 months - 57 months; range, 2 months - 306 months). Of those patients, only 7 (31 %) had post-HSCT reports describing worsening demyelination, while the majority (n = 12, 52 %) demonstrated stable scans. Four patients (17 %; Patients ID2, ID3, ID24, and ID33) had recognizable improvement in white matter disease over time.

Thirty-nine patients (98 %) had at least one pre-HSCT NCV report available for review (Fig. 5). In 36 (92 %) of these patients, neuropathy was present before transplant. Twenty-one patients (54 %) had at least one post-HSCT study beyond day +60, with a median time to last follow-up of 24 months (IQR, 12 months – 78 months; range, 3 months – 306 months). Of this group with long-term post-transplant NCV follow-up, 16 (76 %) demonstrated worsening neuropathy.

### Neuropsychologic, adaptive behavior functioning and quality-of-life outcomes

**Fig. 6:**
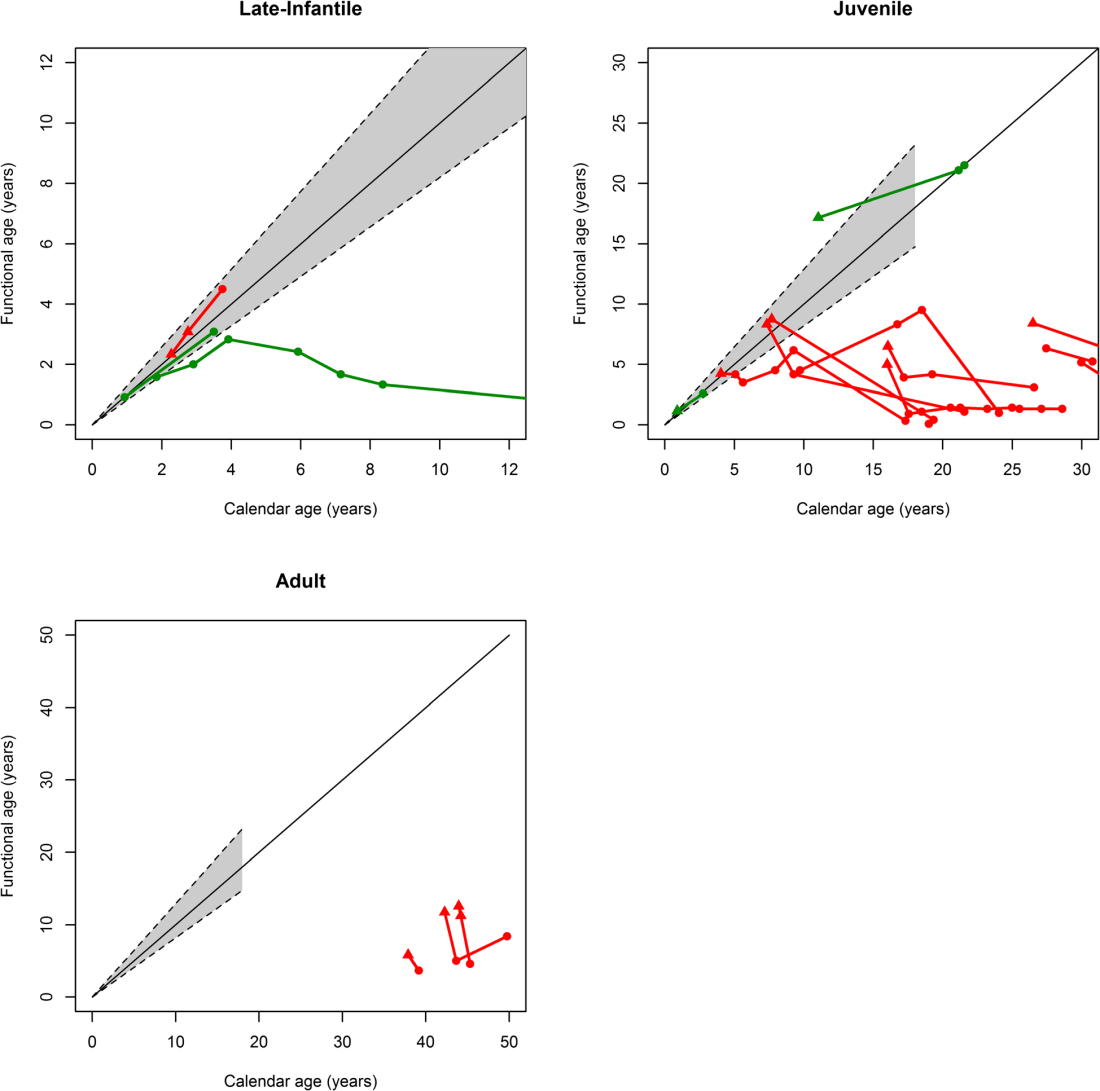
Vineland Adaptive Behavior Scale (VABS) scores over time. Serial trends in composite VABS over years for all long-term survivors after HSCT are shown by MLD subtype. A triangle represents a pre-HSCT score while a circle represents a post-HSCT score. Green indicates patients pre-symptomatic at the time of HSCT. Red indicates patients symptomatic at the time of HSCT. Calendar and functional age equivalence in years is shown by the solid diagonal line, with the dashed lines representing two standard deviations from the mean

**Fig. 7:**
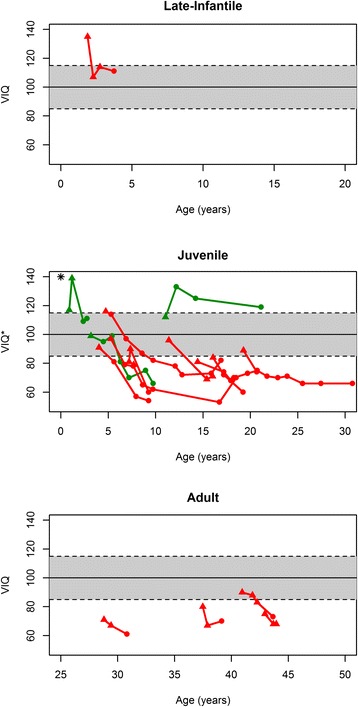
Intelligent Quotient (IQ) Trends over time for MLD subtypes. Trends in IQ scores over time for all long-term survivors after HSCT are shown by MLD subtype. A triangle represents a pre-HSCT score while a circle represents a post-HSCT score. Green indicates patients who were pre-symptomatic at the time of HSCT. Red indicates patients who were symptomatic at the time of HSCT. All but two of the scores shown (both for one J-MLD patient, indicated by asterisk) denote Verbal IQ. The solid line represents a mean IQ score of 100 and the dashed lines are two standard deviations from the mean

Seventeen patients were evaluable for cognitive and/or adaptive behavior functioning over time. VABS data generally reflected significant decline in adaptive behaviors in the immediate months to years after HSCT for all subtypes (Fig. 6). Though all 3 evaluable LI-MLD patients demonstrated normal-for-age adaptive behavior at baseline, the lone patient with extensive follow-up showed significant eventual functional decline. The majority of evaluable J-MLD and A-MLD patients demonstrated adaptive behavior functional impairment at baseline, and most continued to show decline. A similar trend of post-transplant decline, though perhaps slightly more blunted, was seen for VIQ across all subtypes (Fig. 7). Numerical scores were mostly greater than zero across all subsets on the CBS for 12 evaluable survivors, suggesting a favorable quality-of-life (Table 3). Times-to-loss of common ADLs for these same patients are shown in Table 4. When asked, “Are you satisfied with your decision to have your child undergo BMT for MLD?” all 11 parental respondents answered, “Yes” at a median 13 years, 3 months post-HSCT. One surveyed parent declined to respond.

**Table 3:** Cornell-Brown Scale (CBS) quality-of-life scores following HSCT for MLD

ID	MLD Subtype	Age (y,m)^a^	Post-HSCT follow-up (y,m)	Mood-related signs	Ideational disturbances	Behavioral disturbances	Physical signs	Cyclic functions	Total
3	LI	19,11	8,2	6	0	1	-1	-2	4
4	J	34,11	28,2	8	0	4	0	5	17
9	J	17,4	10,5	7	0	-1	-3	2	5
10	J	24,1	12,10	7	3	-1	0	0	9
16	J	22,11	12,3	5	6	-2	-1	1	9
17	J	19,4	5,3	5	0	-2	0	3	6
19	J	19,0	10,5	-5	1	-5	-2	-2	-13
22	J	21,7	9,0	3	3	3	2	-2	9
27	J	26,7	9,0	7	2	1	0	3	13
32	J	45,6	14,10	8	0	5	4	6	23
43	J	21,2	1,6	4	5	6	-1	0	14
39	A	49,9	5,7	0	2	0	0	3	5

Total and sub-domain scores for evaluable long-term survivors using the CBS. More negative (less than zero) scores indicate decreasing quality-of-life; more positive (greater than zero) scores indicate increasing quality-of-life. *LI* Late-Infantile, *J* Juvenile, *A* Adult, ^a^ Age when survey administered, *y* Year, *m* Month

**Table 4:** Age at loss of common activities of daily living (ADLs) following HSCT for MLD

ID	MLD Subtype	Current age^a^	Post-HSCT follow-up^a^	Dressing oneself^a^	Feeding oneself^a^	Meal preparation^a^	Toileting^a^	Self-hygiene^a^	School participation^a^	Work/Volunteer participation^a^	Independent living^a^
3	LI	19,11	8,2	—	—	—	—	—	—	—	—
4	J	34,11	28,2	—	13,0	—	—	—	21,0	26,0	—
9	J	17,4	10,5	—	5,0	—	—	4,0	13,0	—	—
10	J	24,1	12,10	5,10	6,6	—	6,6	7,0	+	—	—
16	J	22,11	12,3	8,4	10,11	—	8,0	8,0	7,0	—	—
17	J	19,4	5,3	9,0	9,0	—	6,0	8,0	+	—	—
19	J	19,0	10,5	8,6	8,6	8,0	8,6	8,6	17,6	—	—
22	J	21,7	9,0	+	+	+	+	+	+	+	+
27	J	26,7	9,0	16,1	16,1	16,1	15,0	+	21,0	—	—
32	J	45,6	14,10	30,0	35,0	19,0	22,0	28,0	17,0	16,0	22,0
43	J	21,2	1,6	+	+	+	+	+	19,0	+	19,0
39	A	49,9	5,7	+	+	42,6	41,8	+	EC	41,8	41,8

^a^ All ages and post-HSCT follow-up times represented as years, months; *LI* Late-Infantile, *J* Juvenile, *A* Adult, *EC* Education completed, — Never achieved ADL, + Currently performing ADL

### Outcome comparisons between MLD sibling pairs

Five MLD sibling pairs were evaluable for some or all of the following outcomes: death from disease progression, gross motor function, expressive language function, adaptive functioning, and independent performance of ADLs (Table 5) Three familial pairs (ID3/sib3, LI-MLD; ID4/sib4, J-MLD; and ID10/sib10, J-MLD) consisted of one transplanted (within our cohort) and one non-transplanted sibling. One pair (ID22/ID27, J-MLD) consisted of two sisters both transplanted within our cohort (one symptomatic and the other pre-symptomatic at HSCT). The final pair (ID17/sib17, J-MLD) consisted of two siblings both transplanted, one within our cohort (symptomatic at HSCT) and the other at another center (pre-symptomatic at HSCT).

**Table 5:** Comparison of outcomes between LI-MLD and J-MLD sibling pairs

Pair (ID)	MLD Subtype	Age at HSCT (Sx)	GMFC-MLD^a^						ELFC-MLD^a^				ADL^b^								VABS^c^ Age Equivalent	Age at Follow-Up (Deceased)
			M1	M2	M3	M4	M5	M6	E1	E2	E3	E4	Dress	Feed	Meal	Toilet	Hyg.	Sch.	Work	Ind.		
A (3)	LI	0,8 (No)	—	—	1,3	1,6	2,0	2,3	2,6	2,9	3,0	5,5	—	—	—	—	—	—	—	—	0,1	19,11
A (3sib)	LI	No HSCT	1,4	1,9	2,0	2,2	2,3	2,10	1,4	1,7	1,7	1,7	—	—	—	—	—	—	—	—	N/A	(4,6)
B (4)	J	4,10 (Yes)	3,0	12,0	12,0	12,0	12,0	24,0	3,0	5,0	18,0	+	—	13,0	—	—	—	21,0	26,0	—	1,7	34,11
B (4sib)	J	No HSCT	4,0	4,0	5,0	5,0	5,0	5,0	4,0	4,0	5,0	5,0	—	4,6	—	4,0	—	9,0	—	—	N/A	(9,3)
C (10)	J	5,8 (Yes)	5,0	7,2	7,9	8,0	9,0	+	6,8	8,0	+	+	5,10	6,6	—	6,6	7,0	+	—	—	1,0	24,0
C (10sib)	J	No HSCT	5,0	5,6	6,8	7,0	7,4	7,10	5,6	6,0	6,8	7,2	5,3	5,10	—	5,10	5,10	9,0	—	—	N/A	(18,0)
D (17)	J	7,8 (Yes)	6,0	7,0	9,0	9,7	12,0	13,4	6,0	9,7	12,6	15,0	9,0	9,0	—	6,0	8,0	+	—	—	0,5	19,4
D (17sib)	J	4,3 (No)	13,0	+	+	+	+	+	14,0	+	+	+	+	+	—	+	+	+	—	—	X	15,2
E (27)	J	16,1 (Yes)	16,0	16,0	16,0	16,0	16,0	+	16,0	+	+	+	16,1	16,1	16,1	15,0	+	21,0	—	—	3,0	26,6
E (22)	J	11,1 (No)	+	+	+	+	+	+	+	+	+	+	+	+	+	+	+	+	+	+	21,6	21,6

Ages expressed as years, months; *sib* Sibling not in our HSCT cohort, *LI* Late-Infantile, *J* Juvenile, *Sx* Symptoms present at transplant, *HSCT* Hematopoietic stem cell transplantation, ^a^ Age in years, months at entry into respective level, see Fig. 1 for GMFC-MLD and ELFC-MLD level definitions, ^b^ Age at loss of independent performance of common activities of daily living, *Dress* Dressing oneself, *Feed* Feeding oneself, *Meal* Meal preparation, *Toilet* Toileting, *Hyg* Self-hygiene, *Sch* School participation, *Work* Work/volunteer participation, *Ind* Independent living, ^c^ Vineland Adaptive Behavior Scales (VABS) composite functional age equivalent score at the calendar age of follow-up, *N/A* Data not applicable, + Loss of skill or function has not yet happened, — Has never achieved skill or function, *X* Data not available

## Discussion

This retrospective, single-center review of 40 patients undergoing allogeneic HSCT for MLD is the largest to date. In this study, we aimed to characterize survival and long-term functional outcomes associated with transplantation. The estimated survival at 5 years post-HSCT was 59 %. Survival did not depend on the MLD subtype classification of the patient, nor did it depend upon the presence of symptoms at the time of transplant. In general, previous studies and case reports have documented improved survival outcomes for transplants performed pre-symptomatically or in those with older age at disease onset [7–9, 11, 12]. However, our data suggest that allograft source may play a more significant role*.* Not surprisingly, a trend towards improved survival was seen for those who received HLA-matched sibling marrow grafts. For patients receiving unrelated donor grafts, survival following UCB transplantation appeared favorable as has been previously reported [7]. Most deaths in our cohort occurred from treatment-related complications within the first year of transplant and comparatively few patients died due to progressive MLD. It is difficult to know whether the relatively high incidence of TRM at 6 months (23 %) is dependent to any degree upon the diagnosis of MLD itself. Importantly, a significant fraction of the cohort was treated in an era when HSCT was riskier, regardless of the underlying disease necessitating transplant. With advances in donor-recipient HLA typing and matching, pathogen detection, antimicrobial therapies, busulfan targeting and allograft availability, it is likely that a similar cohort transplanted in the current era would demonstrate less TRM.

Five patients underwent RIC transplantation according to existing institutional protocols available at the time of treatment. While no TRM was observed for these 5 patients, 2 (40 %) experienced autologous hematopoietic recovery. Even for those who demonstrated donor engraftment, an essential question that cannot be answered in this analysis due to insufficient numbers of evaluable long-term survivors is whether the agents and doses employed in transplant conditioning might impact long-term neurologic outcomes. While a rational argument can be made for the use of CNS-sparing regimens for transplantation of neuropathic metabolic disorders, recent pre-clinical data suggest that HSCT conditioning using CNS penetrating agents, in particular high-dose busulfan, improves donor-derived microglial engraftment which may then be essential for metabolic cross-correction within the CNS [31, 32]. It is perhaps noteworthy that all fatal veno-occlusive disease of the liver (n = 3) in our cohort occurred following busulfan-based conditioning, as MLD can be associated with abnormalities of the biliary system [3]. However, this complication may be expected to be less frequent in the era of therapeutic busulfan monitoring, as data from a more modern MLD transplant cohort is notable for an absence of morbidity from veno-occlusive disease [7].

It is difficult to assess whether transplantation provided a survival advantage compared to natural history for patients within our cohort who did not die from transplant-related causes. Perhaps the largest analysis for untreated MLD survival is provided by a recent literature meta-analysis of 98 LI-MLD, 78 J-MLD, and 127 A-MLD patients [33]. In that report which recognized variability in the “definition” of disease subtypes, the estimated J-MLD survival probability was 50 % at approximately 10 years following MLD symptom onset. Among 16 J-MLD patients in our cohort who did not die from transplant-related causes, 2 have died from progressive MLD at 8 years and 5 years following initial disease onset. Fourteen J-MLD patients survive at a median most recent follow-up of 14.5 years following first symptoms (or from when symptoms would be anticipated based on proband sibling history). Comparison of LI-MLD and A-MLD is difficult owing to relatively small numbers within our cohort.

Long-term neurologic function remains a highly relevant outcome following any therapeutic intervention for MLD. Despite this, relatively few robust, prospective, longitudinal natural history descriptions exist for MLD. Biffi *et al* provided perhaps the most thorough such analysis with their assessment of 26 primarily LI-MLD and J-MLD patients in which they catalogued performance on detailed motor and neuropsychological scales over time [34]. The retrospective nature of our analysis did not allow for such high-resolution longitudinal assessments of our cohort. However, investigators with the German LEUKONET group have recently developed and applied simpler scales to describe the decline of gross motor and expressive language function in large, untreated LI-MLD and J-MLD cohorts [20–22]. Importantly, their GMFC-MLD and ELFC-MLD tools were designed for either prospective or retrospective assessment of an individual patient over time.

For this analysis, we used detailed longitudinal clinical neurology notes or, when possible, recent parental telephone interviews to assess individual performance on the GMFC-MLD and ELFC-MLD scales for long-term survivors. Only two surviving patients in our transplanted MLD cohort fit late-infantile classification as employed by the LEUKONET natural history study, One of these LI-MLD patients (ID3), pre-symptomatic at transplant, showed rapid decline similar to natural history expectations as described by the LEUKONET group. The other (ID2), also pre-symptomatic at transplant, demonstrated more protracted motor decline post-HSCT compared to that reported in the large LEUKONET LI-MLD cohort (Fig. 3) [20]. Similarly, Brazilian investigators documented a relatively large LI-MLD natural history cohort, but included all children who became symptomatic before the age of 48 months. When extrapolated to the GMFC-MLD scale, reported mean ages at entry to motor levels 3, 5 and 6 were 26, 27 and 25.6 months, respectively, for their untreated “late-infantile” patients [17]. In contrast, when considering all four long-term survivors in our transplanted cohort who met this definition for “late-infantile” MLD applied to the Brazilian cohort (ID2, 3, 4 and 9) the ages to entry into the same motor levels were 70, 86, and 137 months, respectively. These findings suggest that HSCT may attenuate the steep motor decline for some LI-MLD patients, even though all such transplanted patients in our cohort ultimately experienced severe motor dysfunction.

Only one long-term surviving LI-MLD patient in our cohort (ID3) was evaluable for expressive language function compared to existing natural history description by LEUKONET. She showed a similar trend compared to untreated patients with perhaps modest prolongation of expressive language function [22]. When extrapolated to the ELFC-MLD tool, the large Brazilian “late-infantile” cohort appeared to enter level 4 at a mean age of 25.4 months [17]. For three evaluable long-term survivors in our transplanted cohort meeting the same definition of “late-infantile” applied in the Brazilian analysis, the mean age at entry into level 4 was 74.5 months (with one patient, ID4, still expressing single word ideas at 34 years of age). Again, these findings suggest that HSCT might modestly blunt the rapid expressive language deterioration in untreated very early-onset MLD, even as all such transplanted patients in our cohort ultimately experienced severe expressive language dysfunction.

As expected given their propensity to have a slower natural history decline, our J-MLD patients showed better preservation of motor and expressive language function following HSCT when compared to LI-MLD counterparts . A relatively large number of long-term J-MLD survivors (n = 13) were evaluable for gross motor and expressive language functioning at a median age of 23 years at the most recent assessment. Of this J-MLD group, 7 patients, all symptomatic at the time of transplant, demonstrated motor decline following HSCT similar to that described by J-MLD natural history cohorts [5, 17]. Interestingly, relative preservation of expressive language was seen for these same patients at the same time. In contrast, other J-MLD patients, particularly those who were pre-symptomatic or only had mild deficits at transplantation, demonstrated gradual or no loss on the GMFC-MLD and ELFC-MLD scales.

The relative preservation of skills and increased variability of response to HSCT in J-MLD may be due to the greater phenotypic variation within this subtype. Therefore, when comparing performance on the LEUKONET scales between the transplanted and reported non-transplanted J-MLD groups, it may be most useful to assess the age of entry into early levels of motor and expressive language dysfunction which would represent more subtle disability. While 2 J-MLD patients in the cohort continue to show no evidence of motor dysfunction at last follow-up, 11 have entered into motor levels 1 and 2 (Fig. 1) at mean ages of 139 and 167 months, respectively. In contrast, the LEUKONET group reported median entry for untreated J-MLD into the same levels at 64.5 and 91 months, respectively [20]. Comparison with the small number of J-MLD patients reported in the Brazilian natural history study also suggests superior motor performance for transplanted J-MLD patients in our cohort [17].

Expressive language function of the transplanted J-MLD group also appeared better than reports for untreated counterparts. While the LEUKONET group reported 50 % of their untreated J-MLD cohort having first evidence of language decline (E1-50 %) at approximately 90 months of age, our surviving transplanted cohort of 13 reached E1-50 % at age 105 months [22]. Furthermore, LEUKONET data revealed 50 % of the J-MLD natural history cohort experienced a total loss of expressive language (E4-50 %) at age 150 months [22]. In contrast, in our transplanted cohort only 4 of 13 evaluable J-MLD patients (31 %) have fully lost expressive language at an average age of 147 months. Importantly, the mean age at most recent follow-up for the remaining 9 transplanted J-MLD patients with functioning level E3 or better is 287 months. Overall, the aggregate motor and expressive language function over time of evaluable J-MLD patients in our transplanted cohort appears to be favorable when compared to previous natural history reports.

Neuroradiographic and neurophysiologic outcomes following HSCT for our cohort generally mirror those reported by others [35, 36]. Lack of access to consistent, primary source data and images precluded our ability to provide longitudinal quantitative description. By qualitative assessment, most patients demonstrated relatively stable brain MRI white matter disease over time. Some patients, particularly in the LI-MLD group, showed improvement following HSCT, though this may have reflected developmental myelination rather than transplant effect. J-MLD patients were most likely to show myelin loss following transplant, but the reason for this is unclear. What is more evident is that the stabilization of MRI findings did not equate to stabilization of peripheral nerve disease. With very few exceptions, NCV scores followed serially throughout the pre- and post-transplant course were conspicuous for a frequent decline over time, even if MRIs performed at the same point in time were similar or better than previous exams (Fig. 5). These collective findings appear to support previous reports and speculations that ARSA secretion by hematopoietically-derived cells of donor origin may more favorably impact central nervous demyelination compared to peripheral nervous system disease [15, 37, 38].

Long-term adaptive behavior functioning and cognitive outcome data after HSCT for MLD are sparse in the medical literature. In our cohort, most long-term survivors had adaptive behavior functioning data captured by the composite VABS score across multiple pre- and post-transplant time points. An inherent advantage of this tool is its reliability in assessing function remotely. Indeed, by parental telephone survey we were able to evaluate 12 long-term survivors in our cohort for current adaptive behavior functioning at a median post-HSCT follow-up of more than 13 years. Although some LI-MLD and J-MLD patients initially displayed adaptive behavior functioning trajectories within a normal range, almost all patients eventually plateaued and then regressed. All evaluable A-MLD patients in the cohort were symptomatic at the time of HSCT and showed relatively stable, although abnormally low, adaptive behavior functioning over time. In contrast to adaptive behavior functioning, longitudinal VIQ appeared to demonstrate less dramatic decline over time for evaluable patients. This finding perhaps also supports relative sparing of central nervous system function, as compared to peripheral nervous system function, following transplant for MLD.

As parents and clinicians consider the utility of HSCT for MLD, they may wish to weigh quality-of-life and daily functioning aspects of patients’ post-transplant courses. Tables 3 and 4 show intriguing data characterizing these outcomes. In this study we used the Cornell-Brown Scale, as it has been designed for proxy (parent/caregiver) assessment of quality-of-life in patients with dementing disorders. Of 12 evaluable long-term survivors, 11 had positive (greater than zero) total scores suggesting a favorable quality-of-life. Furthermore, many J-MLD and A-MLD patients retained the ability to independently perform a wide array of ADLs many years after transplant. Although no robust published data addressing these aspects of untreated MLD patients exist for comparison, these findings may provide more concrete evidence which parents might utilize in making the difficult decision to have their child with MLD undergo transplant. We ultimately asked parents if they were satisfied with their decision to have their child undergo HSCT for MLD. All 11 respondents (100 %) answered affirmatively at a median follow-up of over 12 years following transplant. Interestingly, over half of these responding parents had another child with MLD, many of whom were either not transplanted (due to advanced disease at diagnosis) or underwent transplantation but died due to treatment-related complications.

Marked variation in MLD phenotypes, even within a subtype classification, have greatly hampered the ability to generalize outcomes following treatment. Although intra-familial disease behavior can also vary, it may be less likely to do so. As such, sibling cohort comparisons might allow for optimal assessment of intervention efficacy [8, 12, 39, 40]. Therefore, we described outcome data for all available sibling pairs in which at least one sibling underwent transplant in our cohort (Table 5). Of 5 total dyads, 3 consisted of one treated and one untreated sibling; in all cases, the treated patient has survived their deceased sibling by a significant margin. Expressive language and ADL function were notably superior for all transplanted patients as well. For one LI-MLD pair (A), our transplant cohort member experienced more rapid motor decline compared to her non-transplanted sibling. The reason for this is unclear but may stem from transplant-related toxicity as the decline was immediately following HSCT. It should be noted, however, that this patient demonstrated prolonged preservation of expressive language function compared to her untreated sibling and is still alive nearly 15 years longer than she might presumably be without transplant. For the sibling pairs in which both were transplanted, the pre-symptomatic siblings continue to demonstrate significantly greater retention of function compared to their symptomatic counterparts, again reflecting the importance of transplant in delaying disease progression. In one such sibling dyad, J-MLD pair E, both sisters were transplanted in our cohort. While both patients survive to date, patient ID22 (pre-symptomatic at HSCT) has shown a dramatically superior clinical course characterized by normal-for-age motor, expressive language, ADL, and adaptive functioning. Although she experiences seizures, at age 21 years she is pursuing a university degree. In contrast, her sister, symptomatic at transplant, showed relatively significant decline over the same period. In the other such dyad, J-MLD pair D, the symptomatic familial proband (ID17) underwent transplant in our cohort, while her brother was transplanted while pre-symptomatic at another center. He continues to demonstrate superior function in motor, expressive language and ADL performance when compared to his sister. In summary, these sibling pair data suggest utility in HSCT for MLD and highlight the dramatic benefit that HSCT may provide when performed pre-symptomatically in J-MLD.

While our study provides a relatively robust characterization of various outcomes following HSCT for MLD, there are several recognized limitations. First, owing to the relatively high prevalence of the *ARSA* pseudodeficiency allele, it is important to acknowledge that low ARSA activity alone is not sufficient to diagnose MLD. Exclusion of other rare demyelinating disorders (with coincidental ARSA pseudodeficiency carriage) is typically achieved by the demonstration of hyper-excretion of accumulating substrate (urine sulfatide assessment), pathognomonic histopathology on nervous tissue biopsy, or/and presence of causative *ARSA* mutations on molecular analysis [30]. Very few patients in our cohort had evaluable histopathologic or *ARSA* mutation data for confirmation of MLD diagnosis. All 40 patients included in our analysis had either primary or secondary source documentation of elevated urine sulfatide excretion to implicate MLD as causative of their low ARSA activity and personal or family history of leukodystrophy. As well, patients for whom only secondary source data exists for confirmation of MLD diagnosis are cautiously included in this analysis.

The span of time encompassed in this cohort, nearly three decades, carries with it certain inherent analytic risks and biases. Some data, especially before electronic medical records were widely available, were not as readily collected or evident in paper charts. Yet all attempts were made to track down any data through all resources available. While quantifiable assessment of longitudinal changes in the neuroradiographic and neurophysiologic burdens of MLD are ideal, the lack of retrospective access to consistently obtained, primary source data relegated our analysis to a qualitative one. We were also limited in terms of longitudinal data by difficulties in contacting family members of survivors. However, over half of the families were reached and were willing to participate, some of them decades out from their transplant. Since MLD is a very rare disease and it is even rarer to present in time to be a candidate for transplant, this participation was encouraging and important for analysis of long-term outcomes. The potential exists for recall bias in regards to the retrospective assessment of GMFC-MLD, ELFC-MLD and ADL skills. Yet we recognize that the exact timing of when these abilities were lost is less important than the general trends of skill retention. It must also be noted that all of these skills gradually diminish over time rather than abruptly change which can limit precision. Still, existing natural history data has similarly relied on retrospective parental assessments [20, 22].

Though our data add to existing evidence suggesting utility in HSCT for MLD, it is also clear from both this and previous analyses that transplantation is generally not expected to fully abrogate disease manifestations. And though it would be intriguing to formally assess the effects of graft source, conditioning regimen, age, symptom status and degree of donor hematopoietic engraftment on functional outcomes following transplant for MLD, the small number of evaluable patients in our cohort along with relative homogeneity of outcomes precluded such formal analysis. Still, the limits of HSCT are particularly evident in LI-MLD, even when the child is treated prior to clinically evident disease. Clearly, novel treatment strategies are needed that could be used independently of transplantation or in association with it. A recent report documented the utility of genetically corrected autologous hematopoietic stem cell therapy in a small MLD case series; larger systematic gene therapy studies are ongoing [41]. And although broad-population newborn screening is attractive for the purpose of very early intervention, several unique aspects of MLD present significant challenges to identifying those newborns with true “disease” [42].

## Conclusion

In summary, we report outcomes of the largest single-center cohort of patients who have undergone HSCT for MLD. Our analysis highlights both the potential benefits, as well as limitations, of HSCT for MLD across all three clinical subtypes. While our data strongly suggest efficacy from early, pre-symptomatic transplant in later-onset MLD phenotypes, it also suggests objective and perceived benefits for long-term survivors across all MLD subtypes. While most patients ultimately experienced neurologic decline following transplant, HSCT appears to more favorably impact the trajectory of natural progression of cognitive compared to motor dysfunction. Treatment with HSCT should be carefully considered for patients with MLD, as investigations into other treatment modalities aimed at improving outcomes continue [41, 43, 44].

## References

[1] von Figura K, Gieselmann V, Jaeken J, Scriver C, Beaudet A, Sly W, Valle D (2001). Metachromatic Leukodystrophy. The Metabolic and Molecular Bases of Inherited Diseases, Eighth Edition.

[2] Jeon S-B, Yoon H, Park S-H, Kim I-H, Park E (2008). Sulfatide, a major lipid component of myelin sheath, activates inflammatory responses as an endogenous stimulator in brain-resident immune cells. J Immunol.

[3] Gieselmann V (2008). Metachromatic leukodystrophy: genetics, pathogenesis and therapeutic options. Acta Paediatr Suppl.

[4] Shapiro EG, Lockman LA, Balthazor M, Krivit W (1995). Neuropsychological outcomes of several storage diseases with and without bone marrow transplantation. J Inherit Metab Dis.

[5] MacFaul R, Cavanagh N, Lake BD, Stephens R, Whitfield AE (1982). Metachromatic leucodystrophy: review of 38 cases. Arch Dis Child.

[6] Shapiro EG, Lockman LA, Knopman D, Krivit W (1994). Characteristics of the dementia in late-onset metachromatic leukodystrophy. Neurology.

[7] Martin H, Poe M, Provenzale J, Kurtzberg J, Mendizabal A, Escolar M (2013). Neurodevelopmental outcomes of umbilical cord blood transplantation in metachromatic leukodystrophy. Biol Blood Marrow Transplant.

[8] Solders M, Martin DA, Andersson C, Remberger M, Andersson T, Ringdén O (2014). Hematopoietic SCT: a useful treatment for late metachromatic leukodystrophy. Bone Marrow Transplant.

[9] Kapaun P, Dittmann RW, Granitzny B, Eickhoff W, Wulbrand H, Neumaier Probst E (1999). Slow progression of juvenile metachromatic leukodystrophy 6 years after bone marrow transplantation. J Child Neurol.

[10] Görg M, Wilck W, Granitzny B, Suerken A, Lukacs Z, Ding X (2007). Stabilization of juvenile metachromatic leukodystrophy after bone marrow transplantation: a 13-year follow-up. J Child Neurol.

[11] Kidd D, Nelson J, Jones F, Dusoir H, Wallace I, McKinstry S (1998). Long-term stabilization after bone marrow transplantation in juvenile metachromatic leukodystrophy. Arch Neurol.

[12] Cable C, Finkel R, Lehky T, Biassou N, Wiggs E, Bunin N (2011). Unrelated umbilical cord blood transplant for juvenile metachromatic leukodystrophy: a 5-year follow-up in three affected siblings. Mol Genet Metab.

[13] Solders G, Celsing G, Hagenfeldt L, Ljungman P, Isberg B, Ringdén O (1998). Improved peripheral nerve conduction, EEG and verbal IQ after bone marrow transplantation for adult metachromatic leukodystrophy. Bone Marrow Transplant.

[14] de Hosson LD, van de Warrenburg BPC, Preijers FWMB, Blijlevens NMA, van der Reijden BA, Kremer HPH (2011). Adult metachromatic leukodystrophy treated by allo-SCT and a review of the literature. Bone Marrow Transplant.

[15] Krivit W, Peters C, Shapiro EG (1999). Bone marrow transplantation as effective treatment of central nervous system disease in globoid cell leukodystrophy, metachromatic leukodystrophy, adrenoleukodystrophy, mannosidosis, fucosidosis, aspartylglucosaminuria, Hurler, Maroteaux-Lamy, and Sly syndromes, and Gaucher disease type III. Curr Opin Neurol.

[16] Bredius RGM, Laan LAEM, Lankester AC, Poorthuis BJHM, van Tol MJD, Egeler RM (2007). Early marrow transplantation in a pre-symptomatic neonate with late infantile metachromatic leukodystrophy does not halt disease progression. Bone Marrow Transplant.

[17] Artigalás O, Lagranha VL, Saraiva-Pereira ML, Burin MG, Lourenço CM, van der Linden H (2010). Clinical and biochemical study of 29 Brazilian patients with metachromatic leukodystrophy. J Inherit Metab Dis.

[18] Przepiorka D, Weisdorf D, Martin P, Klingemann HG, Beatty P, Hows J (1995). 1994 Consensus conference on acute GVHD Grading. Bone Marrow Transplant.

[19] Filipovich A, Weisdorf D, Pavletic S, Socie G, Wingard J, Lee S (2005). Diagnosis and staging working group report. Biol Blood Marrow Transplant.

[20] Kehrer C, Blumenstock G, Gieselmann V, Krägeloh-Mann I, Leukonet G (2011). The natural course of gross motor deterioration in metachromatic leukodystrophy. Dev Med Child Neurol.

[21] Kehrer C, Blumenstock G, Raabe C, Krägeloh Mann I (2011). Development and reliability of a classification system for gross motor function in children with metachromatic leucodystrophy. Dev Med Child Neurol.

[22] Kehrer C, Groeschel S, Kustermann-Kuhn B, Bürger F, Köhler W, Kohlschütter A (2014). Language and cognition in children with metachromatic leukodystrophy: onset and natural course in a nationwide cohort. Orphanet J Rare Dis.

[23] Wechsler D (2003). Wechsler Intelligence Scale for Children-Fourth Edition (WISC-IV).

[24] Roid G (2003). Stanford-Binet Intelligence Scales.

[25] Bayley. Bayley Scales of Infant and Toddler Development. 2006. 33620792

[26] Sparrow. Vineland Adaptive Behavior Scales, “Survey Form Manual”, Second Edition. 2005.

[27] Ready RE, Ott BR, Grace J, Fernandez I (2002). The Cornell-Brown scale for quality of life in dementia. Alzheimer Dis Assoc Disord.

[28] Kaplan E, Meier P (1958). Nonparametric estimation from incomplete observations. J Am Stat Assoc.

[29] Lin DY (1997). Non-parametric inference for cumulative incidence functions in competing risks studies. Stat Med.

[30] Rafi MA, Coppola S, Liu SL, Rao HZ, Wenger DA (2003). Disease-causing mutations in cis with the common arylsulfatase A pseudodeficiency allele compound the difficulties in accurately identifying patients and carriers of metachromatic leukodystrophy. Mol Genet Metab.

[31] Wilkinson FL, Sergijenko A, Langford-Smith KJ, Malinowska M, Wynn RF, Bigger BW (2013). Busulfan conditioning enhances engraftment of hematopoietic donor-derived cells in the brain compared with irradiation. Mol Ther.

[32] Capotondo A, Milazzo R, Politi LS, Quattrini A, Palini A, Plati T (2012). Brain conditioning is instrumental for successful microglia reconstitution following hematopoietic stem cell transplantation. Proc Natl Acad Sci U S A.

[33] Mahmood A, Berry J, Wenger DA, Escolar M, Sobeih M, Raymond G (2010). Metachromatic leukodystrophy: a case of triplets with the late infantile variant and a systematic review of the literature. J Child Neurol.

[34] Biffi A, Cesani M, Fumagalli F, Del Carro U, Baldoli C, Canale S (2008). Metachromatic leukodystrophy - mutation analysis provides further evidence of genotype-phenotype correlation. Clin Genet.

[35] van Egmond M, Pouwels PJW, Boelens J-J, Lindemans C, Barkhof F, Steenwijk M (2013). Improvement of white matter changes on neuroimaging modalities after stem cell transplant in metachromatic leukodystrophy. JAMA Neurol.

[36] Groeschel S, í Dali C, Clas P, Böhringer J, Duno M, Krarup C (2012). Cerebral gray and white matter changes and clinical course in metachromatic leukodystrophy. Neurology.

[37] Peters C, Steward CG, Program NMD, Registry IBMT, Working Party on Inborn Errors ErBMTG (2003). Hematopoietic cell transplantation for inherited metabolic diseases: an overview of outcomes and practice guidelines. Bone Marrow Transplant.

[38] Koç ON, Day J, Nieder M, Gerson SL, Lazarus HM, Krivit W (2002). Allogeneic mesenchymal stem cell infusion for treatment of metachromatic leukodystrophy (MLD) and Hurler syndrome (MPS-IH). Bone Marrow Transplant.

[39] Krägeloh Mann I, Groeschel S, Kehrer C, Opherk K, Nägele T, Handgretinger R (2013). Juvenile metachromatic leukodystrophy 10 years post transplant compared with a non-transplanted cohort. Bone Marrow Transplant.

[40] Pridjian G, Humbert J, Willis J, Shapira E (1994). Presymptomatic late-infantile metachromatic leukodystrophy treated with bone marrow transplantation. J Pediatr.

[41] Biffi A, Montini E, Lorioli L, Cesani M, Fumagalli F, Plati T (2013). Lentiviral hematopoietic stem cell gene therapy benefits metachromatic leukodystrophy. Science.

[42] Barcenas M, Suhr TR, Scott CR, Turecek F, Gelb MH (2014). Quantification of sulfatides in dried blood and urine spots from metachromatic leukodystrophy patients by liquid chromatography/electrospray tandem mass spectrometry. Clin Chim Acta.

[43] Batzios S, Zafeiriou D (2012). Developing treatment options for metachromatic leukodystrophy. Mol Genet Metab.

[44] Patil S, Maegawa GHB (2013). Developing therapeutic approaches for metachromatic leukodystrophy. Drug Des Devel Ther.

